# Cardamonin Inhibits Metastasis of Lewis Lung Carcinoma Cells by Decreasing mTOR Activity

**DOI:** 10.1371/journal.pone.0127778

**Published:** 2015-05-21

**Authors:** Pei-Guang Niu, Yu-Xuan Zhang, Dao-Hua Shi, Ying Liu, Yao-Yao Chen, Jie Deng

**Affiliations:** Department of Pharmacy, Fujian Provincial Maternal and Child Health Hospital, Fuzhou, Fujian, China; Peking University Cancer Hospital & Institute, CHINA

## Abstract

The mammalian target of rapamycin (mTOR) regulates the motility and invasion of cancer cells. Cardamonin is a chalcone that exhibits anti-tumor activity. The previous study had proved that the anti-tumor effect of cardamonin was associated with mTOR inhibition. In the present study, the anti-metastatic effect of cardamonin and its underlying molecule mechanisms were investigated on the highly metastatic Lewis lung carcinoma (LLC) cells. The proliferation, invasion and migration of LLC cells were measured by MTT, transwell and wound healing assays, respectively. The expression and activation of mTOR- and adhesion-related proteins were assessed by Western blotting. The in vivo effect of cardamonin on the metastasis of the LLC cells was investigated by a mouse model. Treated with cardamonin, the proliferation, invasion and migration of LLC cells were significantly inhibited. The expression of Snail was decreased by cardamonin, while that of E-cadherin was increased. In addition, cardamonin inhibited the activation of mTOR and its downstream target ribosomal S6 kinase 1 (S6K1). Furthermore, the tumor growth and its lung metastasis were inhibited by cardamonin in C57BL/6 mice. It indicated that cardamonin inhibited the invasion and metastasis of LLC cells through inhibiting mTOR. The metastasis inhibitory effect of cardamonin was correlated with down-regulation of Snail and up-regulation of E-cadherin.

## Introduction

Metastasis is the primary cause of death in most cancer patients. The process of metastasis consists of a series of sequential, interrelated steps. Tumor cells detach from the primary tumor is the first step of metastasis [1]. E-cadherin mediates calcium-dependent intercellular adhesion. It maintains the cell-to-cell adhesion and decreases the separation of cancer cells from cancer tissues. The down-regulation of E-cadherin is a sign of poor prognosis, correlating with invasion and metastasis in multiple carcinomas [2, 3]. In addition, the deficiency of E-cadherin is one of the earliest steps in the epithelial-mesenchymal transition (EMT). Snail is a zinc-finger transcription factor. It binds to the 5’-CACCTG-3’ sequence of E-cadherin promoter and represses the transcription of E-cadherin. Snail induces EMT by suppressing the adhesion proteins such as E-cadherin and matrix metalloproteinases (MMPs), and their expression correlates inversely with the degree of cancer differentiation [4]. Several studies have shown that down-regulation of E-cadherin mediated by Snail results in metastasis [5, 6].

The mammalian target of rapamycin (mTOR) is a central regulator of cell growth, proliferation, differentiation and survival. Recent studies have demonstrated that mTOR also plays a critical role in regulating the motility, invasion and metastasis of cancer cells [7, 8]. The expression of E-cadherin is regulated by mTOR and its downstream target ribosomal S6 kinase 1 (S6K1) [9]. Activation of mTOR decreases the level of E-cadherin by up-regulating Snail and Slug in human ovarian cancer cells [10]. S6K1 is responsible for tumor metastasis through the induction of EMT, and this tumorigenic activity is associated with the ability of S6K1 to repress the expression of E-cadherin [11]. Moreover, activation of S6K1 duplicated the motility and invasion induced by hepatocyte growth factor, suggesting that S6K1 was involved in regulating invasion and motility of cancer cells. Rapamycin is a specific mTOR inhibitor. It inhibits cancer invasion and metastasis in various experimental metastatic models [12, 13]. Therefore, mTOR inhibitors could suppress the invasion and metastasis of cancer cells through increasing the cell-cell adhesions.

**Fig 1:**
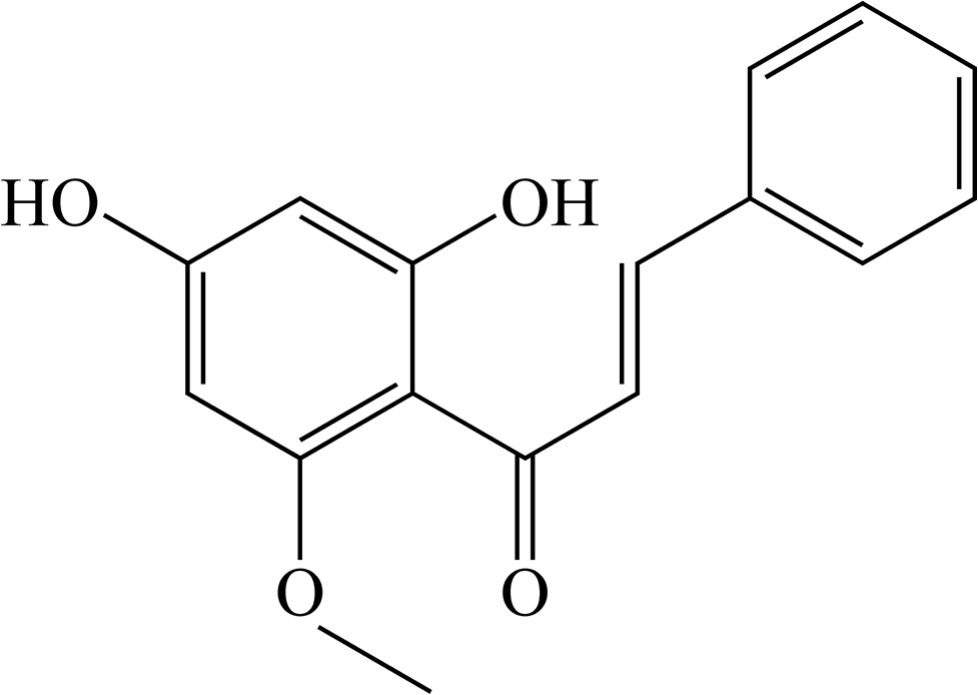
Chemical structure of cardamonin.

*Alpinia katsumadai* Hayata (Zingiberaceae) is a commonly used traditional medicinal plant in China and Korea. Cardamonin is the main flavonoid that derived from the seed of *Alpinia katsumadai* (Fig 1). Previous studies have demonstrated that cardamonin exhibits antiproliferation activity in various cancer cells [14]. Our studies had shown that cardamonin inhibited the proliferation of vascular smooth muscle cells in vitro/vivo and ameliorated insulin resistance through inhibiting the phosphorylation of mTOR and its downstream targets [15, 16]. Furthermore, we found that cardamonin inhibited the proliferation and induced apoptosis of non-small cell lung cancer cells (A549) by decreasing activity of mTOR. More recently, our results showed that cardamonin might directly interact with mTOR [17]. However, whether cardamonin plays a role in preventing metastasis is unknown.

In this study, the effects of cardamonin on the proliferation, invasion and of the Lewis lung carcinoma (LLC) cell line were studied. It was proved that cardamonin inhibited the progression of cancer by several mechanisms including inhibition of proliferation, and repression of migration and metastasis. Our data also showed that cardamonin inhibited the metastatic potential of LLC cells in vitro/vivo through the regulation of E-cadherin expression, which was partial mediated by mTOR.

## Materials and Methods

### Materials

High-glucose DMEM media were purchased from Gibcol (Grand Island, NY, USA). Cardamonin was isolated from the dried seeds of *Alpinia katsumadai* Hayata. (Anhui, China). The purity of cardamonin is > 99% in HPLC analysis. Rapamycin, bovine serum albumin and MTT were from Sigma (St Louis, MO, USA). The 10 mM solution of cardamonin and 100 μM solution of rapamycin were prepared in DMSO, stored at -20°C, and then diluted as needed in cell culture medium. Rabbit antibodies against mTOR, p-mTOR (Ser2448), S6K1, p-S6K1 (Thr389), E-cadherin, Snail and the secondary antibody (anti-rabbit IgG, HRP-linked antibody) were from Cell Signaling Technology (Boston, MA, USA). Antibody against actin was from Santa Cruz Biotechnology (Dallas, TX, USA). Matrigel was purchased from BD Biosciences (San Jose, CA, USA).

### Cell culture

LLC (catalogue number: CX0189) cells were obtained from Boster (Wuhan, Hubei, China) and cultured in high-glucose DMEM with 10% fetal bovine serum (FBS), 100 U/ml penicillin G and 100 μg/ml streptomycin in an atmosphere of 5% CO_2_ at 37°C.

### Cell viability assay

5 × 103 cells per well were cultured overnight in a 96-well plate. The cells were treated with rapamycin (0.1 μM) and cardamonin (0.1, 1 and 10 μM) in the presence of 5% FBS for 48 h. Cell viability was assessed by MTT assay. The number of surviving cells was assessed by the determination of the A490 nm of the dissolved formazan product after addition of 20 μL MTT solution (0.5 mg/ml) for 4 h.

### Transwell invasion assay

In vitro invasive activity through a gel matrix was examined in 24-well plates. Cells were resuspended at a density of 1 × 10^5^ cells/ml. BD Falcon Cell Culture Inserts (San Jose, CA, USA) were coated with 50 μL of Matrigel and placed in each well. Plate wells were filled with 600 μL of complete medium, and the upper insert were filled with 100 μL of the cell suspension. Treated with rapamycin (0.1 μM) and cardamonin (0.1, 1 and 10 μM) for 24 h, the viable invasive cells that adhered to the lower surface of the filter were fixed using 70% methanol and stained with 0.5% crystal violet. Cells that on the upper surface of the filter were removed by cotton swabs. The cells in the lower level were counted, and compared with the other groups.

### Migration assay

Cells were seeded in 6-well plates and grown into a confluent monolayer. A “wound” was inflicted in the cell layer by scratching the plate with a sterile pipette tip and the cells were washed with PBS and incubated with rapamycin (0.1 μM) and cardamonin (0.1, 1 and 10 μM) in the presence of 5% FBS for 24 h. Cells were photographed at **×** 100 magnification at 0 h and 24 h after incubation. The distance travelled by cells was measured between the two boundaries of an acellular area. The results of the different treatment group were expressed as a ratio to original distance.

### *In vivo* studies

Fifty C57BL/6 mice (6-week-old, 18–22 g) were provided by the Experiment Animal Center of Fuzhou General Hospital of Nanjing Military Command (certificate no. SCXK-2010-0005, Fuzhou, China). All experiments were carried out according to the National Institutes of Health Guide for Care and Use of Laboratory Animals. The authors have received permission from the Animal Care and Use Committee of Fuzhou General Hospital, and this study was approved by the Animal Care and Use Committee of Fuzhou General Hospital. The mice were free access to laboratory rodent chow and water, with an automatically controlled photoperiod of 12 h light per day.

100 μL saline containing 1 × 10^5^ of LLC cells was injected subcutaneously into the axilla of the right forelimb of the C57BL/6 mice. Tumor volume was calculated as (length × width^2^)/2. When the volume of the tumor reached 50 mm^3^, the mice were randomized into six treatment groups: model group, solvent group, rapamycin group (1.12 mg/kg), and 3.5 mg/kg, 7 mg/kg, 10.5 mg/kg of cardamonin group. In the in vivo experiment, cardamonin was dissolved in solvent, which consist of 18% ethanol, 10% Tween-80 and 72% distilled water and bacteria were eliminated by filtration before the drug solution was used. Six mice were set up for each group. The size of the tumor was measured thrice a week. Intraperitoneal injections with 0.9% saline (model group), solvent (V_dH2O_:V_Tween-80_:V_dehydratedalcohol_ = 72:10:18, solvent group) or drugs were performed once a day for 20 days. Then the mice were sacrificed, the tumor was isolated. Lung samples were harvested for further examination. The number of lung metastasis was determined by counting the number of metastatic nodules on the lung surface. Then the lungs were fixed with formalin and embedded in paraffin. H&E staining was performed to detect the lung metastasis.

### Western blotting

Cells were treated with different drugs for 24 h in 10 cm dishes. Cell lysates were prepared by the addition of 100 μL lysis buffer (50 mM Tris-HCl, pH 7.5, 150 mM NaCl, 1% v/v Nonidet P-40, 0.5% v/v sodium deoxycholate and 0.1% SDS), and a mixture of protease and phosphatase inhibitors. The lysis buffer was transferred into the 1.5 ml centrifugal tubes. The lysates were incubated on ice for 30 min with vortex. Then the samples were centrifugated at 14 000 × *g* for 20 min at 4°C. Pellets were discarded and the content of solubilized proteins was determined by the BCA method using the BCA reagent. 50 μg proteins were resolved by SDS-PAGE and electrotransferred onto the polyvinylidene difluoride filters for immunoblotting. After probing with phosphorylation-specific antibodies, the membranes were stripped and reprobed with antibodies against total kinase proteins and actin. Signals were detected by chemiluminescence (ECL Plus detection system) and exposure to X-ray film to produce bands within the linear range.

### Statistical analysis

The data were presented as mean ± SD. Statistical analysis was preformed by one-way ANOVA followed by Tukey's post-hoc test. p < 0.05 was considered as significant.

## Results

### Cardamonin inhibited the viability, invasion and migration of LLC cells

**Fig 2:**
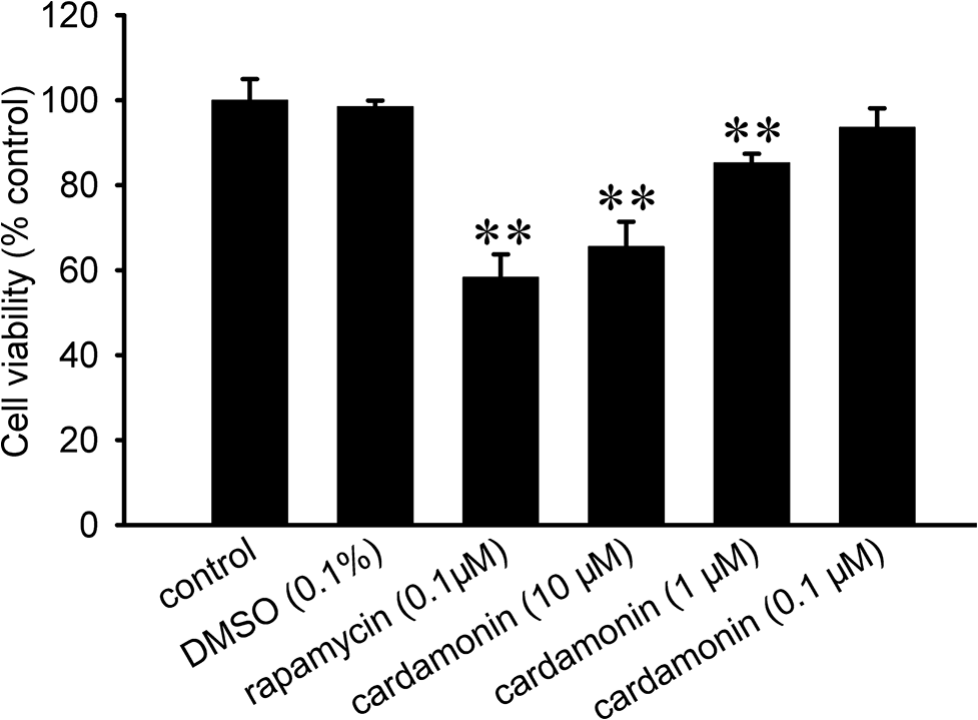
Cardamonin inhibited the proliferation of LLC cells. LLC cells were treated with rapamycin (0.1 μM) and cardamonin (0.1, 1 and 10 μM) for 48 h. The proliferation was analyzed at by MTT method. The cell viability for each group was presented by comparing to the control group. The data were presented as the mean ± SD (*n* = 6). **p < 0.01, compared to the control group.

**Fig 3:**
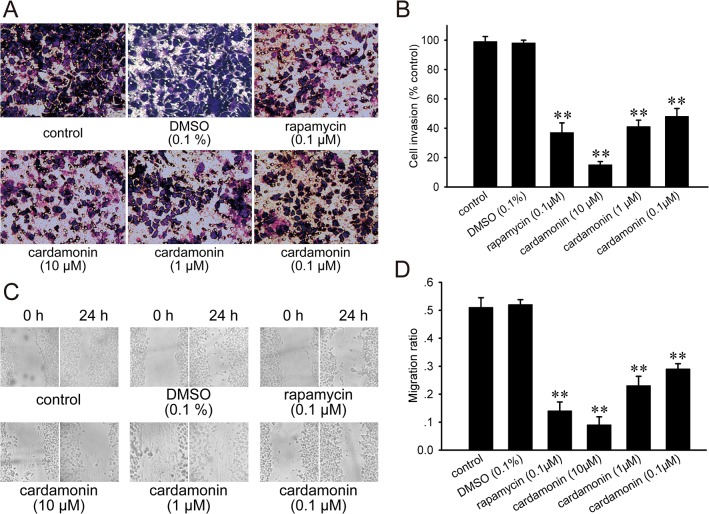
Cardamonin inhibited the invasion and migration of LLC cells. After incubation with different drugs for 24 h, the cells in the top on the chamber were removed. (A) The cells that had migrated onto the bottom of the chamber were stained with crystal violet and photographed at 200 × magnification. (B) The invaded cells were counted, and the invasive ratio for each group was presented by comparing to the control group. The percentage of the penetrated cells for each group was showed as the bar graph. In the migration assay, a wound was inflicted in the cell layer, and then treated with different drugs for 24 h. (C) The denuded zone was photographed at 100 × magnification. (D) The traveled distance of the different treatment group was expressed as a ratio to the original distance. The migration ratio of each group was showed as the bar graph. The data were presented as the mean ± SD (*n* = 5). **p < 0.01, compared to the control group.

To determine whether cardamonin could function as a new therapeutic compound, we investigate the effect of cardamonin on the cell proliferation. The LLC cells were exposed to different concentrations of cardamonin for 48 h in MTT assay. Consequently, the proliferation of LLC was inhibited by cardamonin in a concentration-dependent manner (Fig 2). However, the inhibitory efficacy of each dose of cardamonin was weaker than that of rapamycin. Invasion is an important step of metastasis. The inhibitory effect of cardamonin on the ability of LLC cells to invade a reconstituted extracellular matrix was assessed by transwell chamber. Our result showed that the number of cells that invaded to the lower chamber was significantly decreased (Fig 3A). Compared with control, the levels of invasion were reduced to 14.8%, 40.7%, 47.2% and 36.8% upon cardamonin and rapamycin treatment, respectively (Fig 3B). To further test the influence of cardamonin on the migration on LLC cells, the scratch assay was implemented. As shown in Fig 3C, a gradual increase of cells in the denuded zone was observed after 24 h in the control and DMSO group. When LLC cells were incubated with cardamonin and rapamycin, the cellular motility were decreased gradually (Fig 3D). The inhibitory effect of invasion and migration was stronger in cardamonin (10 μM) than that in rapamycin (0.1 μM).

### Cardamonin inhibited tumor growth and lung metastasis *in vivo*

**Fig 4:**
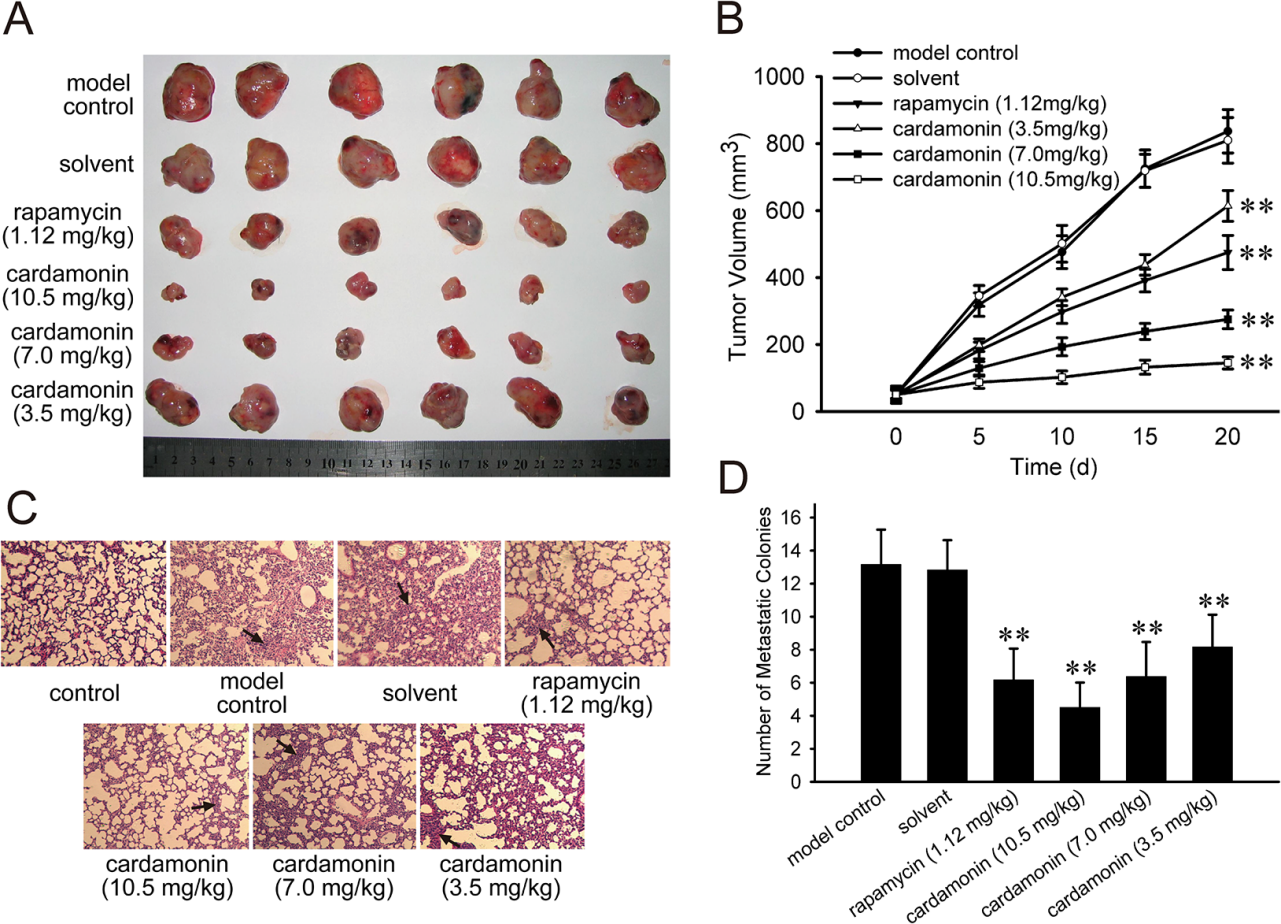
Cardamonin inhibited the tumor growth and lung metastasis of LLC cells in vivo. After 20 days of treatment, the mice were sacrificed and the tumors were isolated. The number of lung metastasis was determined by counting the number of metastatic nodules on the lung surface. (A) Tumors in different groups. (B) The initiation and growth of tumors were determined by measuring the average tumor volume. (C) Representative lung tissue sections were stained with H&E and photographed at 100 × magnification. (D) The number of lung surface metastases formed by LLC cells in each group. The data were presented as the mean ± SD (*n* = 6). The control group represents normal C57BL/6 mice without treatment. The model control group represents C57BL/6 mice that injected s.c. with Lewis lung carcinoma cells and treated with physiological saline.**p < 0.01, compared to the control group.

To further examine the in vivo growth and metastasis inhibitory effect of cardamonin, we employed a organ-selective metastasis experimental model. The LLC cells were transplanted s.c.in C57BL/6 mice and the tumor grows locally and metastasized only to the lung. The size of tumors in the model control, solvent, rapamycin, and cardamonin treated groups were shown in Fig 4A. The tumor growth inhibition ratios were 46.4% (rapamycin), 84.3% (10.5 mg/kg, cardamonin), 71.2% (7.0 mg/kg, cardamonin) and 31.6% (3.5 mg/kg, cardamonin), respectively (Fig 4B). Next, we investigated the effect of cardamonin on the lung metastasis of LLC cells. The representative histological photomicrographs of lung tissue sections were stained with H&E (Fig 4C). Cardamonin and rapamycin showed lung colonisation inhibition of LLC cells. The mean numbers of metastatic nodules on the lung surface of different groups were shown in Fig 4D. Similar with the in vitro anti-metastatic results, the effect of cardamonin at high dose was more powerful than that of rapamycin.

### Cardamonin attenuated the activation of mTOR and increased the expression of E-cadherin

**Fig 5:**
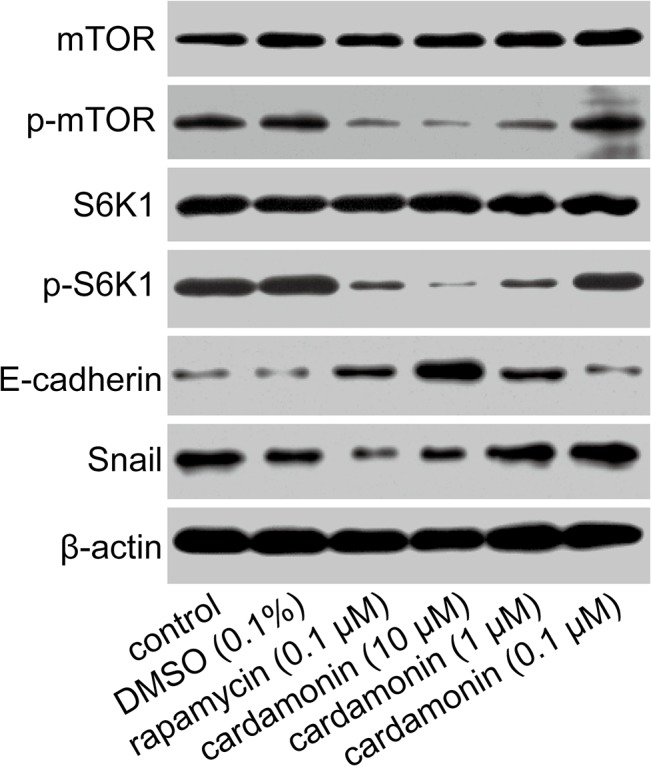
Effects of cardamonin on the phosphorylation of mTOR, S6K1 and the expression of E-cadherin, Snail. LLC cells were treated with different drugs for 24 h, and then the total protein was extracted. Analysis of protein expression of mTOR, S6K1, p-mTOR p-S6K1, E-cadherin and Snail was performed by Western Blot method. Expressions of mTOR, p-mTOR, S6K1, p-S6K1, E-cadherin, Snail and actin were showed as the immunoblot band (*n* = 3).

Western blotting was used to explore the underlying mechanism by which cardamonin exerted its actions. The mTOR pathway is frequently activated in various cancers, which is account for cell growth, proliferation and metastasis. We found that the phosphorylation of mTOR and S6K1 were significantly decreased by cardamonin, indicating that cardamonin effectively inhibited mTOR signal pathway. The expression of E-cadherin is partially regulated by mTOR pathway. mTOR mediated loss of E-cadherin decreases the cellular adhesion, resulting in an increased invasive and metastatic potential. Our results found that cardamonin and rapamycin induced mTOR inhibition was accompanied by increased E-cadherin expression. Then the protein level of Snail, an immediate upstream inhibitory transcription regulator of E-cadherin, was determined to clarify the effect of cardamonin on E-cadherin expression. As expected, the protein amount of Snail was decreased following cardamonin and rapamycin treatment (Fig 5).

## Discussion

Increasing evidence suggests that the mTOR signal pathway plays an important role in regulating cell motility, invasion and metastasis. mTOR is becoming a potential therapeutic target for tumor metastasis. Treated with rapamycin, the cell migration was significantly inhibited in different ovarian cancer cell lines [18]. It had been proved that cardamonin was a potential mTOR inhibitor. In the present study, the inhibitory effects of cardamonin on the migration and invasion of LLC cells were similar to that produced with rapamycin, and cardamonin also suppressed the TPA-induced migration and invasion of HT-1080 cells [19]. Although the effective dose of cardamonin was larger than that of rapamycin, all these results demonstrated that cardamonin exhibited anti-metastatic effects on cancer cells. It provides more evidences for cardamonin function as a new cancer therapeutic compound.

mTOR induces cell motility through decreasing the expression of the focal adhesion proteins, and rapamycin showed an inhibitory effect on cell motility or invasion in endothelial cell lines by regulating the expression of adhesion-associated proteins [1]. Rapamycin blocked the transforming growth factor-β1 (TGF-β1) induced loss of E-cadherin expression in the human tubular epithelial cells in a dose-dependent manner [20]. In mesothelial cells, rapamycin showed a mild protective effect on the EMT, as it increased E-cadherin and decreased the expression of α-SMA as well as that of Snail induced by TGF-β [21]. Furthermore, rapamycin inhibited cell migration of human umbilical vein endothelial cells by inhibiting the expression of the zinc-finger transcription factor, which directly repressed the transcription of E-cadherin [22]. In addition, mTOR inhibitors increased the expression of E-cadherin and suppressed cell motility by regulating the mTOR/HIF-2α signal pathway in human renal cell carcinoma [23]. These studies suggested that mTOR inhibitors could act as novel therapeutic agents to decrease cancer metastasis.

This study demonstrated that the expression of Snail was decreased by cardamonin, while that of E-cadherin was increased. Moreover, the phosphorylation of mTOR and S6K1 were inhibited by cardamonin. Since S6K1 has been shown to repress the expression of E-cadherin through the up-regulation of Snail [11], we presumed that cardamonin could modulate the expression of adhesion proteins through inhibiting the mTOR signal pathway. MMPs are gelatinases which can disturb cell adhesion by processing the components of cell-cell and cell-extracellular matrix contacts [4]. The expression of Snail accelerates the invasive ability of cancer cells by increasing the activity of MMPs. Interestingly, the expression and activity of MMP-2 and MMP-9 were inhibited by cardamonin [19], and cardamonin down regulated on Snail might account for this phenomenon. It indicates that cardamonin regulates various aspects of adhesion associated proteins. The NF-κB transcription factor is associated with cancer metastasis by modulating EMT related factors including E-cadherin, MMP and Vimentin [24]. Several studies had confirmed that cardamonin attenuated the transcriptional activity and phosphorylation of p65 subunit of NF-κB [25, 26]. We hypothesized that the inhibitory efficacy of cardamonin on E-cadherin expression was associated with dual inhibition on mTOR and NF-κB pathway.

Rapamycin showed an inhibitory effect on the metastases of transplanted tumors models. For example, it markedly inhibited metastasis of colon cancer to the liver when CT-26 adenocarcinoma cells were injected into BALB/c mice [27]. In addition, rapamycin prevented tumor growth and pulmonary metastasis in the human renal cell cancer pulmonary metastasis models. The underlying mechanism appeared to be related to inhibition of cell cycle and reduction of the tumor promoting cytokines vascular endothelial growth factor-A and TGF-β1 [28]. In a non-small cell lung cancer cell metastatic model, rapamycin effectively prevent the formation of distant metastases in the lung [29]. In the current experiments, the growth and lung metastasis of LLC cells were inhibited by both cardamonin and rapamycin. Dramatically, no marked hepatotoxicity and nephrotoxicity were found in rats treated with cardamonin, suggesting that cardamonin had a better safety in vivo than rapamycin [15]. Because rapamycin exerts various adverse reactions during its application, cardamonin might have more clinical values as a potential mTOR inhibitor. Furthermore, cardamonin has nephroprotective effect against cisplatin induced renal injury through suppressing oxidative stress and inflammation in rats [30]. It prompts us to investigate its antitumor effect when combination with cisplatin.

## Conclusions

In summary, these results demonstrated that cardamonin inhibited the progression of cancer cells by several mechanisms including inhibition of proliferation, repression of migration and metastasis. Our data showed that cardamonin inhibited the metastatic potential of LLC cells in vitro/vivo through regulating the mTOR mediated expression of E-cadherin. It suggested that cardamonin might be of value in developing as an anti-cancer agent.
